# A global roadmap for inclusive clinical trials in older adults

**DOI:** 10.1016/j.jnha.2025.100591

**Published:** 2025-06-05

**Authors:** Jorge G. Ruiz

**Affiliations:** Memorial Healthcare System, Hollywood, Florida, United States

Clinical trials are key to advancing medical knowledge and clinical practice. Yet, with the global population aging rapidly, older adults are mostly underrepresented in clinical research. The scoping review by Cesari and a distinguished group of researchers, “Enhancing the Methodology of Clinical Trials in Older People: A Scoping Review with Global Perspective”[1] tackles this persistent gap by systematically identifying and summarizing recommendations to improve clinical trial methodologies in older adults, with a focus on the unique challenges that stakeholders face in low- and middle-income countries (LMICs).

The paper highlights a paradox: older adults are the biggest consumers of healthcare, yet they are often excluded from the very research that informs treatment guidelines. This exclusion means clinical findings are often not relevant or applicable to this vulnerable population. From over 4700 articles, the authors synthesized 1119 methodological insights into 120 actionable recommendations, refined by researchers experienced in LMIC contexts. These are grouped into 13 themes covering all aspects of trial design, implementation and evaluation.

The authors acknowledgement of the various obstacles to participation that older adults encounter—from cognitive and physical impairments to transportation issues, ageism, and a dearth of age-friendly infrastructure—is one of its strongest points. These challenges are particularly noticeable in LMICs, where conducting inclusive and representative trials is frequently made more difficult by a lack of resources. The recommendations in response call for more inclusive inclusion criteria, culturally appropriate tools, improved community involvement, and practical trial designs.

In addition to these 13 formal categories, the recommendations contain several cross-cutting or emergent themes that are essential to the success of clinical trials in older adults, especially in low- and middle-income nations, but are not classified as stand-alone categories. Effective communication and knowledge translation strategies that are adapted to local contexts are among them, as are the following: the ethical duty to ensure continuity of care and appropriate post-trial support; the development of geriatrics and ethical research practices; meaningful engagement of communities and caregivers; cultural and contextual adaptation of tools and processes; and the promotion of inclusion, equity, and diversity. These related topics highlight a comprehensive, participant-centered, and context-sensitive methodology that improves the viability, applicability, and moral integrity of studies involving older adults, particularly in settings with limited funding.

The direct input of expert panels from LMICs is also beneficial to the scoping review, as their viewpoints guarantee that the suggestions are based on real-world experiences. The framework is more applicable and useful in a variety of healthcare systems and sociocultural contexts thanks to its global dimension. However, the authors admit that more in-depth discussions with a larger group of LMIC stakeholders may be necessary, and that future work should concentrate on pilot-testing the suggestions to evaluate their practicality and scalability.

All things considered, "Enhancing the Methodology of Clinical Trials in Older People" represents a welcome and essential development in the field of geriatric and applied aging research. It provides a well-thought-out, internationally relevant set of guidelines that tackle persistent obstacles to older adults' participation in clinical trials. By combining structural direction with equity principles, cultural sensitivity, and stakeholder engagement, this scoping review sets the foundation for more ethical, equitable, and impactful research that better reflects and serves the interests of the world's aging population.

## Declaration of competing interest

The author declares that he has no known competing financial interests or personal relationships that could have appeared to influence the work reported in this paper.

## References

[1] Cesari M., Canevelli M., Zhang W., Thiyagarajan J.A., Azzolino D., Cherubini A. (2025). Enhancing the methodology of clinical trials in older people: A scoping review with global perspective. J Nutr Health Aging.

